# ACE2-independent entry factors for SARS-CoV-2 infection and immune activation

**DOI:** 10.1128/mbio.01897-24

**Published:** 2025-12-22

**Authors:** Yiyu Sun, Lok-Yin Roy Wong, Theresa L. Chang

**Affiliations:** 1 Rutgers School of Graduate Studies 215980 , Newark, New Jersey, USA; 2 Public Health Research Institute 424699 , Newark, New Jersey, USA; 3 Department of Microbiology, Biochemistry and Molecular Genetics, Rutgers New Jersey Medical School 12286, Newark, New Jersey, USA; 4 Center for Virus-Host Innate Immunity, Rutgers New Jersey Medical School 12286, Newark, New Jersey, USA; Albert Einstein College of Medicine, Bronx, New York, USA

**Keywords:** SARS-CoV-2, alternative receptors

## Abstract

Severe acute respiratory syndrome coronavirus 2 (SARS-CoV-2), the causative agent of coronavirus disease 2019 (COVID-19), remains a major public health threat, particularly in vulnerable populations. SARS-CoV-2 spike proteins interact with the human angiotensin-converting enzyme 2 (ACE2) receptor, together with accessory molecules that facilitate viral entry, through its spike receptor-binding domain (RBD). Although ACE2 is the primary receptor required for viral replication, its expression patterns do not fully correlate with viral distribution or tissue pathology. Moreover, SARS-CoV-2 has been shown to infect cells and tissues lacking detectable ACE2 expression. Viral entry via ACE2-independent pathways may also confer resistance to some monoclonal antibodies (Abs) targeting the spike RBD that block ACE2-mediated binding. These observations highlight the potential significance of ACE2-independent entry factors in SARS-CoV-2 infection, particularly in vaccinated individuals with Abs directed against ACE2-dependent viral entry. In this review, we discuss the emerging roles of ACE2-independent entry factors in SARS-CoV-2 infection and the immune responses. These factors include CD147, AXL, CD169/Siglec-1, CD209L, CD209, CLEC4G, ASGR1, LDLRAD3, TMEM30A, TMEM106B, transferrin receptor 1, GPR78, integrin α5β1, KREMEN1, LFA-1, and CD4. While ACE2 remains central to viral replication, ACE2-independent entry appears sufficient to elicit immune responses. Therefore, future investigations are warranted to elucidate the roles of ACE2-independent mechanisms in immune-mediated pathology and viral evolution, independent of immune pressure targeting ACE2-mediated entry in previously infected or vaccinated individuals.

## INTRODUCTION

The coronavirus disease 2019 (COVID-19) pandemic, associated with more than 20 million deaths, was caused by severe acute respiratory coronavirus 2 (SARS-CoV-2) and remains a threat due to the continuous evolution of new variants (1, 2). Vaccinated people remain susceptible to infection by emerging variants (3–6), and between 100 and 1,000 COVID-19-related deaths continue to occur each week in the United States in 2025 (7). Although current SARS-CoV-2 variants are less pathogenic than early strains, co-infection of SARS-CoV-2 and influenza virus can have severe disease outcomes (8, 9). Additionally, the emergence of newly evolved strains with better transmissibility and enhanced immune escape poses an ongoing concern. Therefore, a deeper understanding of the mechanisms underlying SARS-CoV-2 infection is essential for developing more effective preventive and therapeutic strategies to protect vulnerable populations.

SARS-CoV-2 entry is primarily mediated by binding of the receptor-binding domain (RBD) of the SARS-CoV-2 spike protein to the human angiotensin-converting enzyme 2 (ACE2) receptor, the entry mechanism of which has been summarized in an excellent review (10). In virus-producing cells, proprotein convertases such as furin cleave Spike proteins into S1 and S2 subunits (10). The S1 subunit contains the N-terminal domain (NTD) and RBD, whereas the S2 subunit enhances membrane fusion. The RBD is the main target of anti-spike neutralizing antibodies (Abs) induced by the early generation of vaccines and therapeutic interventions (10, 11). Multiple ACE2-dependent accessory proteins including furin (12, 13), transmembrane serine protease 2 (TMPRSS2) and TMPRSS4 (14, 15), trypsin (16), neuropilin-1 (17, 18), cathepsins (19, 20), sialic acid-containing glycolipids (21), cellular heparin sulfate (22), interferon-induced transmembrane proteins (IFITMs) (23–25), and phosphatidylserine (PS) receptor (26) promote SARS-CoV-2 entry in ACE2-expressing cells. Note that ectopically overexpressed IFITMs restrict SARS-CoV-2 entry regardless of ACE2 abundance on cells (23) and TMPRSS2 expression can shift anti-viral IFITM3 activity toward viral enhancement in ACE2-expressing cells (27). Additionally, E64d, a cathepsin protease inhibitor, blocks SARS-CoV-2 entry in cells with or without ACE2 overexpression, suggesting the potential role of cathepsins in ACE2-independent viral entry (28).

ACE2 supports efficient SARS-CoV-2 replication and its expression positively correlates with viral loads (29, 30); however, its expression patterns do not fully correspond to infection profiles in tissues, clinical manifestations, or immune responses (31–33). ACE2 expression is low in the lung and limited to type II alveolar cells (AT2) and ciliated cells (32); however, lung pathology extends beyond these cell types. Moreover, SARS-CoV-2 infects organs or cells lacking detectable ACE2, suggesting the involvement of ACE2-independent entry factors (also referred to as alternative receptors) (34–39). Murine ACE2 cannot efficiently support SARS-CoV-2 infection; however, mice expressing human CD147 or human transferrin receptor (hTfR) become susceptible to SARS-CoV-2 and have clinical symptoms, providing direct evidence of ACE2-independent viral entry (40, 41). The contribution of ACE2-independent SARS-CoV-2 entry factors appears less evident in cells with high ACE2 expression (42), which may account for inconsistent findings regarding their significance (reviewed in reference 43). A recent study revealed distinct roles of ACE2 and ACE2-independent viral entry factors in viral infection and immune response, respectively (44), highlighting the critical role of ACE2-independent entry factors in virus-mediated immune activation. Although direct evidence from human or animal studies remains to be established, immune responses triggered through ACE2-independent viral entry may contribute to viral clearance, immunopathology, or immune escape in vaccinated or previously infected individuals when ACE2-dependent entry is targeted. Indeed, the Alpha, Beta, and Delta SARS-CoV-2 variant spike proteins exhibited differential cell interactions and ACE2 dependence (45–47). Furthermore, ACE2-independent SARS-CoV-2 entry is often resistant to the spike RBD Abs (35, 37, 48, 49) and immune escape variants become resistant to anti-RBD Abs targeting ACE2 binding (50–56). A naturally occurring spike mutation, E484D, enables SARS-CoV-2 to enter cells through ACE2-independent pathways and confers resistance to imdevimab, an anti-RBD Ab used for COVID-19 therapy (37, 49). Interestingly, the heavily mutated Omicron variants maintain strong ACE2 binding but evade several approved therapeutic Abs that target RBD-ACE2 interaction (57, 58). This suggests that mutations in the spike protein preserve ACE2 binding while altering epitopes recognized by neutralizing Abs, thereby enabling Omicron to couple immune evasion with efficient viral entry. The highly transmissible Omicron variants exhibit reduced dependence on TMPRSS2, leading to the use of alternative entry mechanisms with a shift in cell tropism and changes in pathogenesis (59–64). Because SARS-CoV-2 evolves with exceptional speed, a better understanding of the role of ACE2-independent entry factors for SARS-CoV-2 is important for designing better anti-viral strategies to dampen infection and virus-mediated immune activation. We note that some of these proteins also serve as entry factors for other viruses (reviewed in reference 43), potentially influencing pathogenesis in co-infected patients. In this review, we discuss the role of ACE2-independent entry factors in infection and immune responses (Table 1). Entry factors discussed here are identified based on studies using loss- or gain-of-function approaches and viral entry assays in addition to their binding to spike proteins. Production of infectious viral particles is not a required criterion.

**TABLE 1:** ACE2-independent entry factors for SARS-CoV-2^*a*^

Receptor	Viral components	Reference
CD147	NA, anti-RBD mAb resistant No full-length spike or RBD binding RBD	(35) (42, 65) (66)
AXL	PS in virions (ACE2 dependent) NTD	(26) (67)
CD169/Siglet-1	NTD	(68)
CD209L/L-SIGN/CLEC4M	NTD (high affinity), RBD, S2 RBD (N-glycans)	(48) (69)
CD209/DCSIGN/ CLEC4L	Spike trimmer	(70)
CLEC4G/LSECtin	RBD (N-glycans) NTD	(70) (71)
LDLRAD3	NTD	(71)
TMEM30A/CD50A	NTD	(71)
TMEM106B	RBD	(72, 73)
TfR	Spike (high affinity) RBD (low affinity)	(40)
GRP78	Spike protein	(74)
Integrin α_5_β_1_	RBD (RGD motif)	(75)
KREMEN1	RBD (high affinity), NTD	(48)
ASGR1/CLEC4H1	RBD (high affinity), NTD	(48)
LFA-1	NA	(76)
CD4	Spike protein	(77)
TLR1	E—induces immune activation M—not involved in immune activation	(44)
Clec4g (mouse)	RBD (N-glycans)	(70)
CD209c (mouse)	RBD (N-glycans)	(70)

^*a*^ Viral components that interact with receptors are indicated. RBD: receptor-binding domain. NTD: N-terminal domain. PS: phosphatidylserine. RGD motif: arginine-glycine-aspartic acid motif. NA: not available.

## CD147

CD147, also known as *basigin* or EMMPRIN for extracellular matrix metalloproteinase inducer, acts as an alternative receptor for SARS-CoV-2 entry into cells with low or undetectable ACE2 expression (35, 66). CD147 is expressed in epithelial cells, neuronal cells, myeloid cells, and lymphocytes and found in various tissues (78, 79). CD147 is upregulated in subjects who have an increased risk of severe COVID-19 disease. For example, CD147 is elevated in tumor tissues and is involved in modulating the tumor microenvironment and cancer progression (80–86). Its level is higher in obese diabetic adults (87), which may contribute to their higher risk for severe COVID-19.

CD147 serves as a receptor for multiple viruses including measles, HIV, HBV, HCV, SARS-CoV, and Kaposi’s sarcoma-associated herpesvirus (43), binds to spike RBD proteins, and mediates ACE2-dependent and independent SARS-CoV-2 entry (66). Mepolizumab, a humanized CD147 neutralizing monoclonal Ab (mAb), blocks SARS-CoV-2 infection *in vitro* (66). We found that either CD147 blocking mAb or CD147 knockdown suppressed infection of A549 cells, lung epithelial cells expressing low ACE2 (35). Interestingly, neutralizing anti-spike RBD Abs cannot block infection of A549 cells by pseudotyped SARS-CoV-2 viruses (35), suggesting that RBD binding to ACE2 is not involved in CD147-mediated viral entry (35). Conflicting results indicate CD147 did not bind to SARS-CoV-2 spike proteins and plays no role in viral entry in cell lines expressing high levels of ACE2 (42, 65). These discrepancies may reflect differences in the cell lines used (high versus low ACE2 expression), variations in spike protein preparation, or differences in experimental design. Nevertheless, human CD147 knock-in mice (C57BL/6J or NOD *scid* IL2Rgamma^null^) support SARS-CoV-2 infection and exhibit pathology (41, 66), highlighting the importance of CD147 for SARS-CoV-2 infection and pathogenesis.

CD147 plays a role in macropinocytosis (88), an actin-mediated, clathrin-independent endocytic pathway that facilitates viral entry (89). Macropinocytosis plays a critical role in SARS-CoV-2 viral entry in both ACE2-dependent and independent infection (90, 91). In the presence of ACE2, macropinocytosis promotes SARS-CoV-2 viral entry and cell-cell fusion (91). The macropinocytosis inhibitor 5-*N*-ethyl-*N*-isopropyl amiloride (EIPA) inhibits viral entry. Interestingly, overexpression of TMPRSS2 partially blocks the inhibitory effect. Blockade of macropinocytosis regulators including epidermal growth factor receptor, PI3K, RhoA, Rac1, Cdc42, and Pak1 suppresses viral entry mediated by SARS-CoV-2 spike protein but not entry mediated by VSV G (91). Macropinocytosis also plays a critical role in SARS-CoV-2 syncytium formation, facilitating viral spread through cell-cell fusion (91). The FDA-approved drug apilimod, an endocytosis inhibitor, suppresses SARS-CoV-2 infection of lung organoids and A549 cells lacking ACE2, indicating that non-ACE2-mediated viral entry is dependent on macropinocytosis (90). scRNA-seq analysis of SARS-CoV-2-infected human lung organoids showed that viral RNA is detected in numerous cell types in an ACE2-independent manner (90). This analysis also showed that high levels of viral transcripts occurred in cells expressing cathepsin B/L/S and CD147/BSG (90). CD147 promotes cellular entry of human cytomegalovirus strains that express the pentamer complex into epithelial and endothelial cells through macropinocytosis (92). A recent study reported that CD147 facilitates pseudotyped SARS-CoV-2 infection through caveolar/lipid raft- and cytoskeleton-mediated endocytosis, but independent of the clathrin-mediated endocytosis and macropinocytosis in Vero cells (93). ADP-ribosylation factor 6 (Arf6), a key regulator of clathrin-independent endocytosis and of CD147 endocytic recycling, plays a critical role in pseudotyped SARS-CoV-2 viral entry (93). Because both Vero and Huh7 cells express substantial levels of ACE2, the role of CD147 in macropinocytosis-mediated SARS-CoV-2 infection in cells without ACE2 warrants further investigation.

Recombinant protein subunits of spike S1, RBD, and S2 can stimulate macropinocytosis in murine bone marrow-derived macrophages (BMDMs) as determined by internalization of FITC- and TRITC-dextran (70 kDa), the latter of which is pH-independent (94). Spike protein-mediated macropinocytosis is abolished in macrophages in which pH regulation by the Na^+^-H^+^ exchanger 1 (NHE1) is abrogated either by pharmacological inhibition (by amiloride) or by genetic deletion of NHE1 from myeloid cells (*LysmCre^+^ Nhe1*^*f/f*^). SARS-CoV-2 spike protein subunits also induce macropinocytosis in human monocyte-derived macrophages (MDMs) but not human alveolar epithelial cells. Inhibition of PKC, PI3K, and NADH oxidase 2 suppresses spike protein-induced macropinocytosis in murine BMDMs. Intraperitoneal injection of spike protein S1 was found to induce macropinocytosis in peritoneal macrophages elicited by thioglycolate, but no effect was found in similarly treated macrophages from myeloid cell-specific NHE1-deficient mice. In human macrophages, ACE2-independent, S1-induced macropinocytosis is suggested by studies using LL-37, an antimicrobial peptide that blocks the binding of ACE2 to RBD (94). Taken together, these disparate studies indicate that CD147 is not only involved in SARS-CoV-2 viral entry but also contributes to spike protein-mediated macropinocytosis. It remains to be determined what, if any, role CD147 plays in inducing immune activation.

## AXL

AXL (from the Greek word *anexelekto*, which means uncontrolled, also named UFO for its unidentified functions) is a tyrosine-protein kinase receptor in the TAM family (Tyro3, AXL, and Mer), and is important for its oncogenic potential (95, 96). AXL and its ligand GAS6 regulate innate immune responses and are critical in cancer progression and resistance to targeted therapies (96–98). AXL is expressed in CD34^+^ progenitors, peripheral monocytes, bronchial cells, and marrow stromal cells, but not in lymphocytes or granulocytes (99, 100). It is also present in multiple tissues, including gastrointestinal (GI) tract, lungs, reproductive tissues, and trachea, where ACE2 expression is limited to specific cell types (67, 101).

AXL enhances infection by dengue virus, zika virus, Lassa virus, Ebola, Marburg, Hantaan virus, and Andes virus (43). AXL acts as an ACE2-independent entry factor for SARS-CoV-2 but can also promote ACE2-dependent viral entry (26, 67). AXL was identified as an alternative receptor by using tandem affinity purification-mass spectrometry to analyze protein complexes interacting with SARS-CoV-2 spike proteins in pulmonary and bronchial cells (NCI-H1299 and BEAS-2B) that do not express ACE2 (67). AXL has been identified as an ACE2-independent receptor for SARS-CoV-2 based on several lines of evidence: (i) knockdown of AXL in ACE2-negative, AXL-high cells inhibited viral infection; (ii) soluble AXL blocked viral entry; and (iii) ectopic expression of AXL in AXL-negative 293T cells rendered them susceptible to infection (67). AXL interacts with the NTD but not the RBD of the spike protein (67).

AXL or other PS receptors, including Tim1 and Tim4, promote ACE2-dependent SARS-CoV-2 infection in cells co-expressing ACE2 (26). The cysteine protease inhibitor E-64, which blocks endosomal cathepsins, suppresses both ACE2-mediated entry and AXL- or Tim1-mediated enhancement (26). In cells expressing TMPRSS2, AXL or Tim1 does not increase ACE2-mediated entry, and infection is insensitive to E-64, indicating that PS receptors are not required for plasma membrane fusion (26). The AXL inhibitor bemcentinib blocks infection in Vero cells and certain lung cell lines including some with low ACE2 but not in Calu-3 cells with high TMPRSS2 (26). HEK293T cells expressing AXL alone do not support SARS-CoV-2 infection, and NTD-Fc does not bind AXL-expressing cells; rather, AXL and Tim1 interact with virion-associated PS (26). Whether AXL contributes to SARS-CoV-2-mediated immune activation or whether its ligand GAS6 facilitates viral entry remains to be determined.

The GAS6-AXL signaling pathway modulates membrane ruffling and macropinocytosis through actin remodeling, contributing to tumor progression (102, 103). AXL is known to enhance macropinocytosis of Zaire Ebolavirus glycoprotein-mediated viral entry (104). It also mediates the entry of severe fever with thrombocytopenia syndrome virus through a PI3K-PLC-dependent pathway (105). However, the potential role of AXL in mediating SARS-CoV-2 entry through macropinocytosis remains to be determined.

## C-TYPE LECTIN RECEPTORS

C-type lectin receptors CD209L (L-SIGN/CLEC4M), CD209 (DC-SIGN/CLEC4L), and CLEC4G (LESCtin) act as pathogen receptors that modulate immune responses (106–111). CD209L expression is abundant in AT2 and endothelial cells of the lung, liver, and kidney, while CD209 is mainly expressed in dendritic cells and macrophages (107). Both receptors mediate entry of multiple viruses, including Sindbis virus, HIV, Japanese encephalitis virus, Ebola, HCV, influenza A virus, and SARS-CoV-2 (43). HUVEC-TERT cells expressing CD209L but not ACE2 support productive SARS-CoV-2 infection, which is reduced by CD209L knockdown or soluble CD209L and enhanced by overexpression of CD209L or CD209 (33, 69). CD209L binds the SARS-CoV-2 spike NTD, RBD, and S2 domains, with the strongest affinity for the NTD (48, 69). CD209 binds trimeric SARS-CoV-2 spike (70). CD209L-ACE2 interaction facilitates viral entry independently of the C-type lectin domain, and deglycosylation of CD209L enhances spike RBD binding (69). Despite high sequence homology, CD209 and CD209L show distinct expression patterns, with CD209 enriched in IGSF21^+^ cells and CD209L restricted to vascular structures (33). Lempp et al. demonstrated that overexpression of CD209L, CD209, or CD169/Siglec-1, an I-type lectin receptor (see below), in HEK293T cells, which express very low levels of ACE2, promotes VSV-SARS-CoV-2 pseudotyped viruses (33). However, this infection can be suppressed by small interfering RNAs or polyclonal Abs targeting ACE2, suggesting that these lectins act as attachment factors rather than independent entry receptors (33). Authors also demonstrated the critical role of lectins in facilitating *trans* infection in cells lacking ACE2 such as HeLa cells, despite the absence of productive infection. Moreover, the presence of these lectin receptors modulates the activity of neutralizing Abs, especially Abs targeting regions outside of the receptor-binding motif (RBM), which fail to neutralize SARS-CoV-2 in ACE2-overexpressing cells (33). While RBM mAbs showed differential neutralizing effects in *cis* infection depending on Siglec-1 versus CD209L or CD209 in cells with low ACE2, all RBM mAbs blocked *trans* infection of lectin-expressing cells (Siglec-1 or CD209) to ACE2-expressing cells (33). These findings suggest that lectin receptors can impact the efficacy of neutralizing Abs. The role of CD209 in SARS-CoV-2 entry in myeloid cells (macrophages or dendritic cells) and virus-induced immune response remains to be elucidated.

Macropinocytosis facilitates vaccinia virus entry into monocyte-derived DCs but functions independently of C-type lectin receptors, including CD209/DC-SIGN and mannose receptors (112). It also contributes to DC-mediated HIV endocytosis and transmission *in trans*, although the direct role of CD209, a key receptor for HIV capture, in macropinocytosis-driven HIV transmission *in vitro* has not been examined (113). The role of C-type lectin receptors participate in macropinocytosis-mediated entry of SARS-CoV-2 remains to be determined.

CLEC4G (LSECtin) was independently identified as a SARS-CoV-2 receptor in two cell-based screening studies (70, 71). Zhu et al. used a genome-wide CRISPR activation screen and validated CLEC4G as one of three functional ACE2-independent receptors facilitating viral entry (71). CLEC4G is expressed in sinusoidal endothelial cells of the liver, lymph node as well as in thymic dendritic cells, MDMs, and dendritic cells (109). It regulates T-cell functions (110, 114), binds to filovirus and SARS-CoV spike glycoproteins (115), and acts as a receptor for various viruses including Lassa virus, Japanese encephalitis virus, Ebola virus, and Marburg virus (reviewed in reference 43). CLEC4G recognizes N-acetyl-glucosamine-containing glycans and binds to the SARS-CoV-2 spike NTD of SARS-CoV-2 spike proteins (71). Its expression enables SARS-CoV-2 entry in HEK293T cells, while knockdown blocks infection in SH-SY5Y cells, and soluble CLEC4G moderately inhibits infection in Huh7.5 cells (71).

Using a lectin domain library, Hoffmann et al. identified mouse Clec4g and CD209c as high-affinity spike-binding proteins (70). Deglycosylation of spike proteins reduced their binding, and structural modeling showed that CLEC4G interacts with the N343 glycosylation site within the RBD. High-speed atomic force microscopy confirmed multiple CLEC4G and CD209 molecules binding per trimeric spike. Unlike CD209, CLEC4G binding interferes with ACE2-RBD interaction. CLEC4G and CD209c reduced SARS-CoV-2 infection in Vero and Calu-2 cells, whereas ASGR1 had no effect. Note that conflicting data on ASGR1 as an alternative receptor for SARS-CoV-2 have been reported (see below and reference 48). While human CLEC4G and CD209 are shown to act as SARS-CoV-2 alternative receptors (69, 71), compelling evidence for CD209c and mouse Clec4g as ACE2-independent entry factors is not yet available.

## I-TYPE LECTIN RECEPTORS

CD169/Siglec-1 is a sialic-acid-binding I-type lectin that plays a central role in viral recognition and immune activation in myeloid cells. CD169 is expressed in a subpopulation of macrophages in lymphoid tissues and myeloid cells such as dendritic cells and monocytes (111, 116–118). CD169 promotes DC-mediated SARS-CoV-2 transmission to cells expressing ACE2 (33, 119). Interestingly, macrophages capture SARS-CoV-2 through CD169 but do not transmit viruses to other cells (119). Jalloh et al. showed that SARS-CoV-2 enters macrophages through CD169 and the spike NTD is involved (68). Macrophages are not productively infected by SARS-CoV-2. However, abortive virus replication generates viral negative-strand RNA and subgenomic RNAs, which induce pro-inflammatory cytokine expression via retinoic acid-inducible gene I (RIG-I) and melanoma differentiation-associated gene 5 (MDA-5) (68). The link between ACE2-independent viral entry and inflammatory responses in myeloid cells is also reported by Duan et al. (44). SARS-CoV-2 ORF6 proteins from productive ACE2-dependent virus replication block NF-κB signaling, resulting in minimal inflammatory responses in epithelial cells. In contrast, the production of ORF6 from subgenomic RNAs is restricted in myeloid cells lacking ACE2 while SARS-CoV-2 NSP14 translated from viral genomic RNA acts as a positive regulator of NF-kB signaling and contributes to robust inflammatory responses (44). NSP14 exhibits additional functions that shut down host protein synthesis and IFN anti-viral responses (120). In addition, ACE2-independent SARS-CoV-2-mediated immune activation in myeloid cells can be triggered by the activation of TLR1 through SARS-CoV-2 envelope (E) proteins (44). Note that both E and M proteins bind to TLR1, but only E protein induces immune activation (44).

## LDLRAD3, TMEM30A, and TMEM106B

LDLRAD3, TMEM30A, and TMEM106B are membrane-associated proteins involved in intracellular trafficking and transport and were identified as ACE2-independent SARS-CoV-2 entry factors by a genome-wide CRISPR screening (71, 72, 121).

LDLRAD3 is highly expressed in myeloid cells and various tissues (122), enhances E3 ubiquitin ligase activity (123), and acts as a receptor for Venezuelan equine encephalitis virus (124). TMEM30A (CDC50A), a ubiquitously expressed β-subunit of the phospholipid flippase (P4-ATPase), maintains plasma membrane phospholipid asymmetry and the macrophage “eat-me” signal (125–128). It also supports hematopoietic cell survival (129) and, together with NRP2 and CD63, mediates Lujo virus entry (130).

LDLRAD3 and TMEM30A bind to the NTD of SARS-CoV-2 spike proteins (71). Both proteins function as ACE2-independent SARS-CoV-2 receptors, supporting viral entry in ACE2-deficient HEK293T cells and in multiple cell types, as confirmed by loss-of-function analyses. Furthermore, soluble LDLRAD3 inhibited SARS-CoV-2 infection, confirming its role in viral entry (71).

TMEM106B is a lysosomal membrane protein expressed in many cell types and tissues and has been associated with age-dependent neurodegeneration (131, 132). TMEM106B interacts with vacuolar ATPase and microtubule-associated protein 6 to modulate critical lysosomal functions (133, 134). TMEM106B modulates SARS-CoV-2 infection, particularly in ACE2-independent settings, but is dispensable for other coronaviruses including HCoV-229E and HCoV-OC43 (72, 121). In cells lacking ACE2 expression, TMEM106B supports infection by early SARS-CoV-2 isolates (Belgium/GHB-03021, Germany/BavPat1 that emerged at the beginning of the pandemic) and variants including the E484D mutant (28, 49), a variant that has higher infectivity during viral passaging and may contribute to viral resistance against sera from SARS-CoV-2-infected or vaccinated subjects (135). SARS-CoV-2 infection of ACE2-deficient cells can be suppressed by mAbs against TMEM106B (28). TMEM106B promotes spike-mediated fusion but does not affect viral attachment or endocytosis (28). Structural analysis shows that the luminal domain of TMEM106B interacts with the SARS-CoV-2 spike RBD (28). It is proposed that TMEM106B first anchors to the large loop of RBD (aa 471–491) followed by interacting with another loop of RBD (aa 444–451) (73).

Further evidence of the contribution of TMEM106B to ACE2-independent SARS-CoV-2 entry has also been found using SARS-CoV-2 mouse-adapted 1 virus (SARS-CoV-2_MA1_), which contains spike protein-E484D (136). SARS-CoV-2_MA1_ infection in HEK293T cells requires heparan sulfate and endocytic pathways with TMEM106B (136). Although SARS-CoV-2_MA1_ productively infects human brain organoids and K18-hACE2 mouse brains, it does not infect C57BL/6J or *Ifnar^−/−^* mouse brains or lung (136). Notably, TMEM106B-lentivirus transduction of *Ace2^−/−^* mouse lungs does not lead to detectable SARS-CoV-2_MA1_ replication, indicating that TMEM106B does not support SARS-CoV-2_MA1_ replication in *Ace2^−/−^* mouse (136).

## TRANSFERRIN RECEPTOR (TFR)

TfR1 is widely expressed in various cell types and tissues. It mediates the entry of several viruses including New World arenaviruses, parvoviruses, HCV, and human adenoviruses, which utilize TfR1 to gain access to the endosomal compartment (137). TfR mediates SARS-CoV-2 entry in ACE2-independent manner by using loss- and gain-of-function approaches (40). Human TfR binds the SARS-CoV-2 spike protein with high affinity (*K*_*D*_ 2.95 nM). The TfR has a weaker binding affinity to RBD (*K*_*D*_ 43 nM) than to spike proteins, suggesting that the RBD is not the sole interaction domain. Based on sequence alignment, four TfR residues (A529, K531, V532, and K534) may contribute to the TfR-spike protein interaction. Mutation of TfR A529 to I529 results in the loss of TfR-spike interaction (shown by loss of co-immunoprecipitation). Mice expressing human TfR exhibit an increased susceptibility to SARS-CoV-2 infection (40). Inhibition of spike protein-TfR interactions by small peptides and Abs targeting TfR resulted in suppression of SARS-CoV-2 infection in cells (40). Treatment with anti-TfR Abs also reduced viral loads and lung pathology in SARS-CoV-2-infected rhesus macaques compared to untreated animals (40). It remains to be determined whether TfR is involved in virus-mediated immune activation, which may contribute to reduced pathology in infected animals with anti-TfR-Abs.

## GLUCOSE-REGULATED PROTEIN 78 (GRP78)

GRP78, also known as “Binding immunoglobulin protein” (BiP), is a member of the heat shock protein 70 (HSP-70) family and acts on endoplasmic reticulum-residing chaperones to regulate cell functions by controlling protein folding and degradation (138–140). GRP78 plays a critical role in the cellular entry of enveloped viruses including coronaviruses such as MERS-CoV and bCoV-HKU9 by promoting viral attachment and internalization (141, 142) and non-enveloped viruses. GRP78 mRNA is upregulated in peripheral and lung monocytes and macrophages in severely ill COVID-19 patients compared to healthy subjects, whereas ACE2 mRNA and TMPRSS2 mRNA were not detected in these data sets (74). GRP78 binds SARS-CoV-2 spike protein (*K*_*D*_ 55.2 nM) in a similar range as ACE2 (*K*_*D*_ 23.2 nM) (74). Ectopic expression of GRP78 in HEK 293T cells significantly increases binding of SARS-CoV-2 spike protein. Pseudotyped SARS-CoV-2 infection is significantly increased in GRP78 overexpressing-monocytic THP-1 cells compared to THP-1 cells with undetectable ACE2, supporting a role of GRP78 in ACE2-independent viral entry (74). Because GRP78 plays a critical role in cancers and other diseases (143), it may contribute to increased susceptibility and severity of COVID-19 in high-risk subjects.

## INTEGRIN α_5_β_1_

Integrins play a critical role in inflammation (144, 145) and serve as receptors or attachment factors for the entry of a variety of viruses (146, 147). Several integrins, including integrin α_5_β_1_, bind to SARS-CoV-2 spike proteins (reviewed in reference 148). Bioinformatics and structure-based analyses revealed that SARS-CoV-2 RBD contains an arginine-glycine-aspartic acid (RGD) motif, a recognition site for integrin receptors, but this motif is absent in other coronaviruses (148). ATN-161, the ligand-binding inhibitor for integrin α_5_β_1_, inhibits SARS-CoV-2 infection *in vitro* and in k18-hACE2 mice and blocks SARS-CoV-2-spike protein-induced epithelial dysfunction (75, 149, 150). Note that ATN-161 is not a direct RGD-blocking inhibitor. The SARS-CoV-2 spike protein also induces endothelial inflammation through integrin α_5_β_1_ (151). However, published results on the role of integrin α_5_β_1_ as an ACE2-independent entry receptor for SARS-CoV-2 have been inconsistent (152, 153). Knocking down integrin α_5_β_1_ in cells with high levels of ACE2 did not affect SARS-CoV-2 spike protein-mediated viral entry (152). Interestingly, integrin α_5_β_1_ facilitates spike-mediated cell-cell fusion and inflammation in the presence of ACE2 (152). ATN-161 and high RGD mimicking inhibitor MK-0429 do not inhibit the interaction between integrin α_5_β_1_ and spike protein, nor do they inhibit infection and cell fusion (152). Liu and colleagues show that integrin α_5_β_1_ acts as an alternative receptor in the absence of ACE2 when cells are treated with MnCl_2_, a well-known integrin-activating reagent (153). Cilengitide—an RGD analog cyclic pentapeptide that blocks the binding of RBD to purified integrin α_5_β_1_ extracellular domain and to Mn^2+^ activated ACE2^null^ CHO cells expressing integrin α_5_β_1_. SARS-CoV-2 infects Mn^2+^ activated integrin α_5_β_1_ expressing cells in the absence of ACE2, and the infection is sensitive to Cilengitide, adding evidence that integrin α_5_β_1_ acts as an ACE2-independent entry factor for SARS-CoV-2.

Integrin α_5_β_1_ may inhibit ACE2-dependent viral entry (153). In ACE2-expressing CHO-K1 cells, Mn²^+^ activation of integrin α5β1 markedly reduced viral entry, whereas its inhibition by Cilengitide restored ACE2-dependent infection. These findings suggest that integrin α_5_β_1_ and ACE2 mediate mutually exclusive entry pathways. Pull-down assays further indicate that integrin α5β1 can bind the SARS-CoV-2 spike or RBD in the presence of ACE2, though with reduced affinity, likely due to steric interference. Thus, integrin α_5_β_1_ promotes viral entry in the absence of ACE2 but dampens infection when ACE2 is present.

## KREMEN1 AND ASGR1

KREMEN1 and ASGR1 are ACE2-independent SARS-CoV-2 entry factors identified via high-throughput screening of 5,054 human membrane proteins (48). KREMEN1, expressed in brain, reproductive tissues, and skin, regulates Wnt signaling and cell survival (Wnt-independent) (154–156) and mediates entry of coxsackievirus A10 and other human type A enteroviruses (reviewed in reference 43). ASGR1 is a calcium-dependent C-type lectin receptor and is expressed primarily in hepatocytes (157). ASGR1, a hepatocyte-expressed C-type lectin, internalizes asialoglycoproteins and serves as a receptor for hepatitis E virus (157, 158). Both entry factors bind to the SARS-CoV-2 spike RBD and NTD, but not SARS-CoV spike protein (48). They mediate viral entry in ACE2-negative cells and in mice expressing these receptors via lentiviral transduction (48). In ACE2-positive cells, ACE2-neutralizing Abs block infection, whereas in ACE2-negative lines, KREMEN1 or ASGR1 mediates entry. In some cells, such as NCI-H1944 and NCI-H661, both ACE2 and KREMEN1 contribute (48). In human lung organoids, ACE2 dominates entry, but combined blockade of ACE2, KREMEN1, and ASGR1 provides stronger inhibition, highlighting the coexistence of ACE2-dependent and independent infection routes (48).

## LYMPHOCYTE FUNCTION-ASSOCIATED ANTIGEN 1 (LFA-1) AND CD4

Several studies have reported the detection of SARS-CoV-2 in T lymphocytes (CD3^+^, CD4^+^, or CD8^+^ cells) in blood or lung tissues from COVID-19 patients (66, 76, 159, 160). The ability of SARS-CoV-2 to infect CD4^+^ T cells, which express undetectable ACE2, has been demonstrated using replication-competent viruses and VSV-based SARS-CoV-2 pseudotyped viruses (66, 76, 77, 160). Although viral entry of PBMCs or CD4^+^ T cells was sensitive to the inhibitor of TMPRSS2 camostat mesylate (10–50 μM), it is not clear whether the observed inhibition was due to specific anti-viral effects or the cytotoxicity of the compound (77, 160). SARS-CoV-2 infects CD4^+^ primary cells or transformed T cell lines lacking detectable ACE2 (66, 76, 77). Shen et al. showed that SARS-CoV-2-infected T cells from patients express AXL and LFA-1 but low abundance of ACE2, TMPRSS2, ASGR1, KREMEN1, and NRP1 (76). Loss- or gain-of-function studies indicate that LFA-1, but not AXL-1, mediates ACE2-independent infection of transformed T cells (Jurket cell lines) by SARS-CoV-2 (76). Brunetti et al. reported that SARS-CoV-2 RBD or full-length spike proteins interact with the N-terminus domain of CD4-IgG V type proteins with high affinities (*K*_*D*_ 22–27 nM). SARS-CoV-2 infects CD4^+^ T cells via CD4 receptors as demonstrated by using soluble CD4 proteins and anti-CD4 neutralizing antibody to suppress viral infection (77). Additionally, transformed T cell lines with CD4 are highly susceptible to SARS-CoV-2 infection than T cells without CD4. RNA-seq analysis revealed that SARS-CoV-2 infection alters immune response-related pathways and induces gene expression associated with cell stress and death (77). Consistent with these findings, Shen et al. found that SARS-CoV-2 infection induces T cell death, possibly via the ROS-HIF-1a pathway (76).

SARS-CoV-2 infection also upregulates IL-10 expression in CD4^+^ T cells, consistent with elevated IL-10 expression in CD4^+^ T cells from bronchoalveolar lavage fluid or blood of patients with severe COVID-19 compared to those with moderate diseases or healthy subjects, suggesting that infection of CD4^+^ T cells may contribute to immune dysregulation in severe COVID-19 (77).

## POTENTIAL RECEPTORS IN PLATELET ACTIVATION

Platelets contribute to the severity of COVID-19 (161). Activation of platelets by SARS-CoV-2 is well documented despite the presence of ACE2 expression in platelets being controversial (162–165). SARS-CoV-2 induces platelet activation via the binding of spike RBD to TLR4 (164). SARS-CoV-2 infects human platelets and the progenitor megakaryocyte cell line MEG-01, which do not express ACE2 but do express CD147, GRP78, KREMEN1, and ASGR1 (163). Elevated RNA levels of CD147 and KREMEN1 and reduced NRP1 were found in platelets of ICU COVID-19 patients; similar findings were seen in MEG-1 cells 2 h after exposure to SARS-CoV-2, suggesting that these ACE2-independent entry factors may contribute to platelet-associated immunopathology in COVID-19 patients (163). Tang et al. showed that the serum levels of SARS-CoV-2 E proteins were associated with thrombosis in COVID-19 patients (165). SARS-CoV-2 E proteins activate platelets via p38 MAPK/NF-κB pathways and induce thrombosis in mice (165). The membrane protein CD36 mediates E protein-induced platelet activation and thrombosis (165). The role of TLR4 or CD36 in SARS-CoV-2-mediated platelet activation and thrombosis remains to be determined in an infection model.

## CONCLUSIONS AND PERSPECTIVE

**Fig 1:**
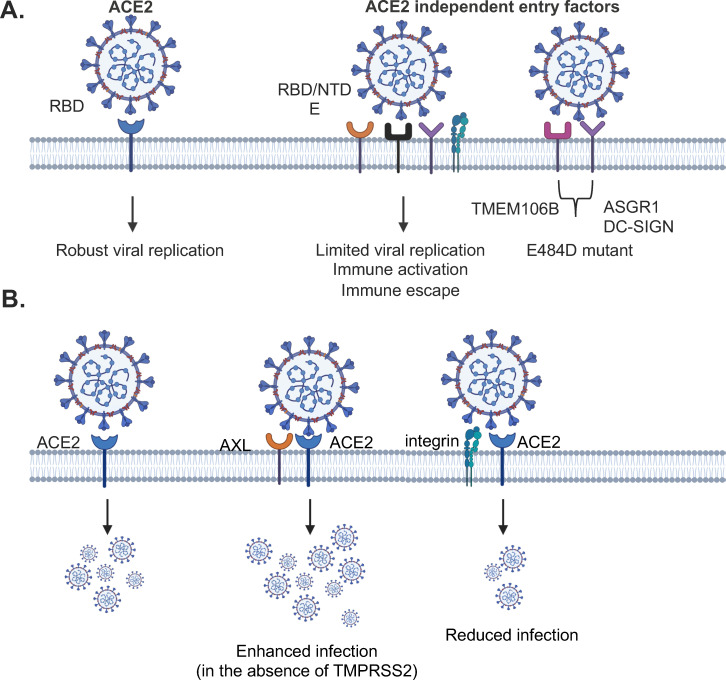
ACE2-independent entry factors for SARS-CoV-2 infection and immune activation. (**A**) ACE2, the primary receptor for the SARS-CoV-2 spike RBD, is essential for efficient viral replication. However, ACE2-independent alternative entry factors (CD147, AXL, C- and I-type lectin, LDKRAD3, TMEM30A, TMEM106B, TfR, GRP78, Integrin α_5_β_1_, KREMEN1, ASGR1, LFA-1, and CD4)—widely expressed across various cell types—can also mediate viral entry, albeit less efficiently. Various domains on the spike proteins and E proteins are involved. The interaction of viruses and alternative receptors (e.g., TMEM106B may occur in endosomal compartments). These alternative receptors may play a crucial role in immune activation, particularly in myeloid cells. Additionally, they can act synergistically to facilitate entry of the SARS-CoV-2 E484D variant that is resistant to antibodies targeting RBD through ACE2-mediated viral entry. (**B**) Alternative receptors can enhance or inhibit ACE2-mediated SARS-CoV-2 entry. The enhancing effect of AXL on ACE2-mediated infection is absent in cells expressing TMPRSS2.

ACE2 is the primary receptor required for robust SARS-CoV-2 replication. However, studies suggest that ACE2-independent entry factors with broader tissue distribution may also contribute to infection and pathogenesis. Further animal and human studies are needed to clarify their roles, particularly in SARS-CoV-2-infected patients and vaccinated individuals with Abs targeting ACE2-mediated viral entry. These ACE2-independent factors may facilitate infection by newly evolved variants, modulate ACE2-dependent viral entry either positively or negatively, and influence immune responses and pathology (Fig. 1). A similar phenomenon is observed in murine coronavirus infection: the neurovirulent MHV-JHM strain, which uses murine CEACAM1a as its receptor, can still infect and spread in the central nervous system of *mCeacam1a*-deficient mice via a CEACAM1-independent mechanism (166), suggesting that the use of ACE2-independent entry factors is not limited to SARS-CoV-2.

The molecular mechanisms underlying SARS-CoV-2 entry and immune activation via ACE2-independent entry receptors in specific cells and tissues remain to be elucidated. The potential involvement of cytoplasmic signaling motifs within these receptors (e.g., CLR, TAM, and CD147) in post-entry events also warrants investigation. Furthermore, unfractionated heparin can block the interaction of SARS-CoV-2 spike proteins with human epithelial cells through an ACE2-independent mechanism (167), highlighting the need to explore the role of heparan sulfate proteoglycans in ACE2-independent viral entry. Clear and detailed descriptions of experimental systems including the presence or absence of ACE2, TMPRSS2, and TMEM106B, which can influence the activity of ACE2-independent entry factors, are essential for generating reproducible results and reconciling discrepancies among studies. Most investigations to date have relied heavily on overexpression of these alternative entry factors in HEK293T cells or on spike proteins fused to other molecules (e.g., Fc tags). Future studies employing pseudotyped and authentic viruses with site-specific spike mutations, in combination with monoclonal Abs targeting defined spike regions, will be critical for delineating the molecular interactions between SARS-CoV-2 and ACE-independent entry factors, particularly in physiologically relevant primary cells and tissues. Spike mutants resistant to monoclonal Abs that block RBD-ACE2 interactions may serve as valuable tools for identifying viral determinants responsible for alternative entry factor usage. For example, ACE2-independent entry of the E484D S mutant pseudotyped lentiviral particles and resistance to imdevimab are mediated through ASGR1 or DC-SIGN with TMEM106B (49). It remains to be determined whether the immune and cell responses by ACE2-independent entry factors contribute to long COVID or infection/pathology in the high-risk groups with elevated levels of some of these receptors. Elucidating how individual ACE2-independent entry factors, acting alone or in combination with ACE2 or other receptors, influence viral infection, virus-mediated immune responses, immune escape, pathogenesis, and disease progression will be essential for developing novel preventive and therapeutic strategies against SARS-CoV-2 and related viruses.
